# Treatment of osteosarcoma with microwave thermal ablation to induce immunogenic cell death

**DOI:** 10.18632/oncotarget.2310

**Published:** 2014-08-04

**Authors:** Zhe Yu, Jie Geng, Minghua Zhang, Yong Zhou, Qingyu Fan, Jingyuan Chen

**Affiliations:** ^1^ Center of Orthopedic Surgery, Orthopedics Oncology Institute of Chinese PLA, Tangdu Hospital, Fourth Military Medical University, Xi'an, Shaanxi, P. R. China; ^2^ Medical Department of Tangdu Hospital, Fourth Military Medical University, Xi'an, Shaanxi, P. R. China; ^3^ Faculty of Military Preventive Medicine, Fourth Military Medical University, Xi'an, Shaanxi P. R. China

**Keywords:** Immunogenic cell death, osteosarcoma, thermal therapy, microwave ablation, cytotoxic T cells

## Abstract

Microwave ablation (MWA) has been used as a classical hyperthermic ablation method for decades with the intention to induce direct killing of tumor cells or modulation of tumor architecture. The purpose of this study was to explore whether MWA induced tumor cell death could generate an immunogenic source of tumor antigens and elicit tumor-specific immune responses, taking an alternative antitumor effects. Three kinds of osteosarcoma cell lines, respectively derived from mice, rats and human, were selected as ablation models. *In vitro* and *in situ* tumor ablation were both performed to detect the “damage-associated molecular patterns” (DAMPs) exposure level. Active ablated products vaccination resulted in complete protection in both mouse and rat tumor-bearing models, which was mediated primarily by vaccine-elicited CD8^+^ T cells. These effector cells functioned by releasing IFN-γ and TNF-α in the presence of target cells, which may trigger FasL-directed cell apoptosis. These data suggest that MWA-processed osteosarcoma cells could be applied to generate specific antitumor effects, especially for *in situ* ablation. Hence, MWA could be used in combination with immunotherapy, especially for patients who have failed chemotherapy or who have limited treatment options.

## INTRODUCTION

Literature concerning the use of high temperatures as treatment for cancer has existed for centuries. Evidence regarding use of hyperthermic ablation for cancers of the liver, breast, lung, and prostate gland has accumulated steadily [1–5]. Various energy sources are available to heat biological tissues: microwave ablation (MWA), radiofrequency ablation (RFA), laser thermal ablation and ultrasonic ablation. MWA and RFA are most commonly used worldwide. During a procedure involving thermal ablation, a thin applicator is guided into the target tumor under image guidance. Energy is then applied to the tissue until temperatures rise to cytotoxic levels (50–60°C). Whether the intent is cure or palliation, the goal of hyperthermic ablation is direct killing of tumor cells or modulation of tumor architecture.

In our center, malignant bone tumors of the extremities and pelvis have been treated by MWA as a classic therapeutic process [6]. When limb-salvage surgery is nearly unfeasible, tumor ablation provides more alternatives for incurable extremity musculoskeletal sarcomas except for the traditional surgical excision [7]. If a bone tumor can be isolated from surrounding normal tissues with an appropriate margin, heat can be applied *in situ* while carefully protecting normal tissues from excessive heat However, in some cases, lesions cannot be separated from adjacent organs. Hence, to avoid injury to adjacent organs, the junction sites between lesions and normal tissues must not experience too high a temperature or too long an ablation time. Usually, the temperature at these junction sites is kept ≤50°C. However, this strategy can be problematic: thermal ablation may not be at a sufficiently high temperature to kill tumor cells and can lead to locoregional recurrence of cancer.

Therefore, in hyperthermic methods such as MWA and RFA, a “gray zone” of ablation is created whereby the most outer margin of ablation contains some living cells. This gray zone is likely to be another source of incomplete ablation, thereby increasing the risk of residual tumor cells or tumor recurrence. Based on clinical data from our research team, locoregional relapse does not occur as frequently as we would expect, so another mechanism of killing of tumor cells may be occurring. Preclinical studies in various tumor models have shown that exposing tumor cells to lethal doses of radiation can elicit cell death while inducing strong antitumor immunity, a process termed “immunogenic cell death” (ICD) [8–10]. Here, we explored the immune responses to MWA-processed tumor cells. In this way, we provided evidence supporting ICD effects induced by MWA during treatment of osteosarcoma.

## RESULTS

### MWA induces time-dependent ICD of mouse, rat, or human osteosarcoma cell lines *in vitro*

First we examined the *in vitro* effect of different times of MWA on the growth, viability, and cardinal signs of ICD in three osteosarcoma cell lines: K7M2 syngeneic to Balb/c mice, UMR106 syngeneic to SD rats, and the human osteosarcoma cell line MG63. Cells were mock ablated (0 min) or ablated for 10, 20 or 30 min. Oxaliplatin (OXP) was used as a positive control to induce ICD [11].

The immunogenic characteristics of this mode of cell death are mediated primarily by molecules called “damage-associated molecular patterns” (DAMPs), most of which are recognized by pattern-recognition receptors. The cardinal signs of ICD are (a) calreticulin (CRT) exposure on the surface of dying cells [12], (b) secretion of high-mobility group box 1 (HMGB1) protein [13], (c) release of adenosine triphosphate (ATP) [14], and most importantly, (d) cell death. DAMPs have a beneficial role in anticancer therapy by interacting with the immune system [15].

**Figure 1:**
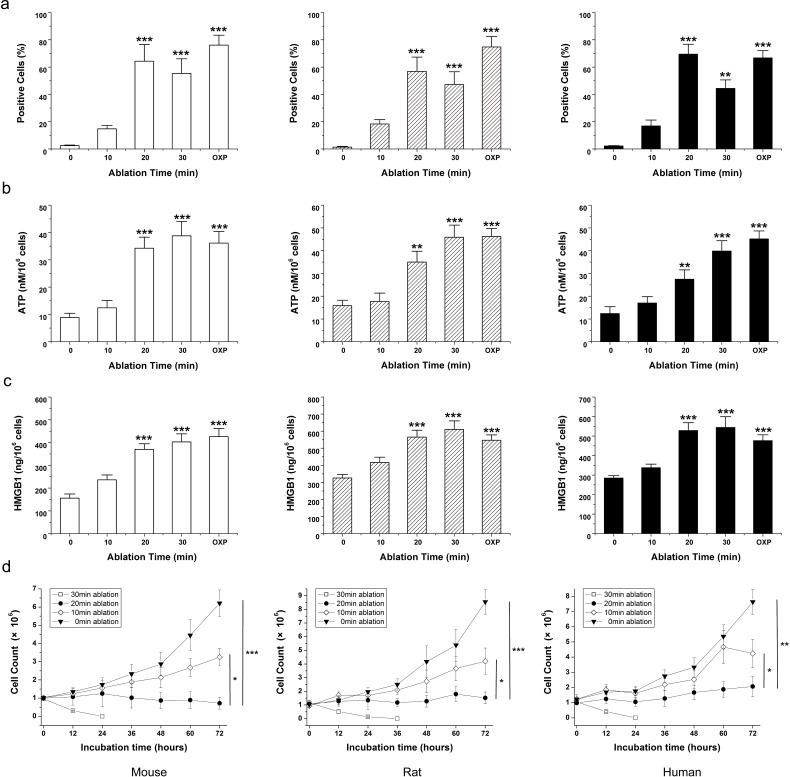
MWA induces a continuum of time-dependent cellular changes related to immunogenic cell death Mouse osteosarcoma (K7M2, open bars), rat osteosarcoma (UMR106, hatched bars), and human osteosarcoma (MG63, black bars) cells were exposed to 10, 20 or 30 min MWA or mock irradiation at 0 min. Oxaliplatin (OXP; 1 mM/mL) was the positive control for ICD. (a) Exposure to MWA for >20 min elicited a significant increase in CRT expression on the surface of ablated tumor cells. (b) In all osteosarcoma cell lines, MWA for >20 min induced significant ATP release and (c) HMGB1 secretion. (d) Cell viability is indicated by the percentage of 7-AAD^−^ cells. Exposure to 20 min or 30 min of MWA significantly decreased cell viability over 72 h relative to controls. Results are the mean ± SEM from three replicate wells. Asterisks denote significance relative to mock-irradiated cells. *P<0.05, **P<0.01, ***P<0.001.

In each cell line, exposure to MWA for 20 min or 30 min showed a significant increase in CRT expression on the surface of ablated tumor cells (Fig. 1a). CRT is a critical component of antigen processing and loading into major histocompatibility complex (MHC)I. Flow cytometric analyses revealed that the highest level of CRT expression on the cell surface appeared in the MWA group for 20 min, which was approximately consistent with that for OXP-treated cells. After 30 min of MWA, CRT exposure on the cell surface should have been sufficient but partial lysis of positive cells could explain the relatively low expression. MWA for 20 min also induced significant release of ATP (Fig. 1b, *P*<0.001) and HMGB1 secretion (Fig. 1c, *P*<0.001) in all types of osteosarcoma cells. Exposure for 20 min or 30 min to MWA significantly decreased cell viability over 72 h in all cell lines relative to controls (*P*<0.0001) (Fig. 1d). These data suggested that MWA induced time-dependent immunogenic alterations in mouse, rat and human osteosarcoma cells ranging from release of DAMPs to cell viability. Also, MWA for 20 min was considered to be a sublethal ablation time that could decrease cell viability and elicit the highest level of CRT expression and considerable release of other DAMPs.

### Protection against tumor challenge by immunization with microwave-ablated tumor cells and supernatants in mice

Exposing mouse osteosarcoma K7M2 cells to microwaves for a sublethal ablation time has been shown to increase surface expression of CRT as well as the release of ATP and HMGB1. Hence, we wished to ascertain if these altered tumor cells and released constituents could work as tumor vaccines and protect animals from fresh challenge by osteosarcoma cells *in vivo*.

In this experiment, mice were immunized with 1×10^6^ ablated tumor cells combined with 1 mL of ablated supernatant injected subcutaneously into the right flank thrice at two-week intervals. On day 0 (2 weeks after the third immunization), vaccinated rats were challenged subcutaneously on the left flank with a lethal dose of tumor cells (1×10^6^, K7M2) and monitored for tumor growth and survival every week.

**Figure 2:**
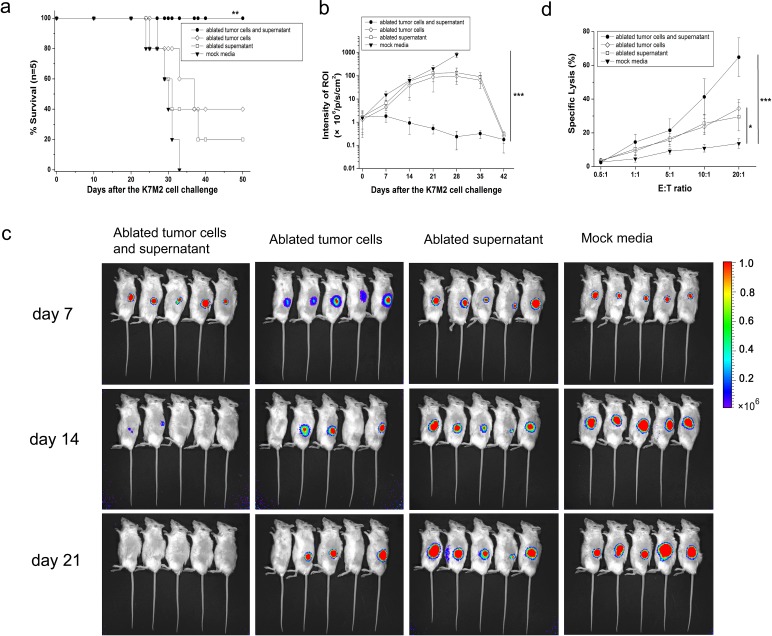
Complete protection of mice against lethal challenge with osteosarcoma cells (a) Survival curve of vaccinated mice and mock media control after tumor challenge. All ablated tumor cells/supernatant-vaccinated mice survived after tumor challenge and appeared to be tumor-free by log-rank test compared with the mock media control group. (b) After a lethal challenge with 1×10^6^ osteosarcoma K7M2 cells, tumor growth was assessed by bioluminescence imaging at day 42 and compared with ablated tumor cells/supernatant-vaccinated mice and mock media control. Data are the mean ± SEM. (c) Representative bioluminescence images of growth of K7M2 tumor cells in vaccinated mice and the mock media control group. Images were acquired at the indicated time points after tumor challenge. (d) Cytotoxicity of splenocytes isolated from vaccinated mice or the mock media control group showed a significant difference. *P<0.05, **P<0.01, ***P<0.001.

All mice immunized with 1×10^6^ ablated tumor cells combined with ablated supernatant (1 mL) were able to reject tumor challenge and remain tumor-free (Fig. 2a). By contrast, immunization with ablated tumor cells alone, ablated supernatant alone, or mock media failed to generate the same level of antitumor protection (*P*<0.01). Based on monitoring of ROIs *in vivo*, ablated tumor cell/supernatant-vaccinated mice proved significantly more efficient than controls at eliminating implanted tumor cells (Fig. 2b, *P*<0.001), which resulted in complete rejection 3 weeks post-challenge, and vaccinated mice remained tumor-free for >6 weeks (Fig. 2c). Splenocytes isolated from ablated tumor cell/supernatant-vaccinated mice showed a strong cytotoxic T cell effect against fresh K7M2 cells *in vitro* that was significantly different from that in the mock media control group (Fig. 2d).

### Protection against tumor challenge by immunization with microwave-ablated tumor cells and supernatants in rats

To confirm further the protection afforded by the “ablated vaccine” against challenge by lethal osteosarcoma cells, we sought to repeat the *in vivo* vaccination and tumor challenge mentioned above in SD rats.

In each group, five rats were immunized with 1×10^7^ ablated tumor cells combined with 1 mL of ablated supernatant, and ablated products were delivered subcutaneously with a needle in a total volume of 1 mL into the groin. Immunization was repeated twice at two-week intervals. On day 0 (2 weeks after the third immunization), 1×10^7^ of UMR106 cells were injected subcutaneously into the left leg. Tumor size was measured every seven days with a caliper. Tumor volume was calculated using the formula:

Tumor volume = π/6 × L × W × H.

**Figure 3:**
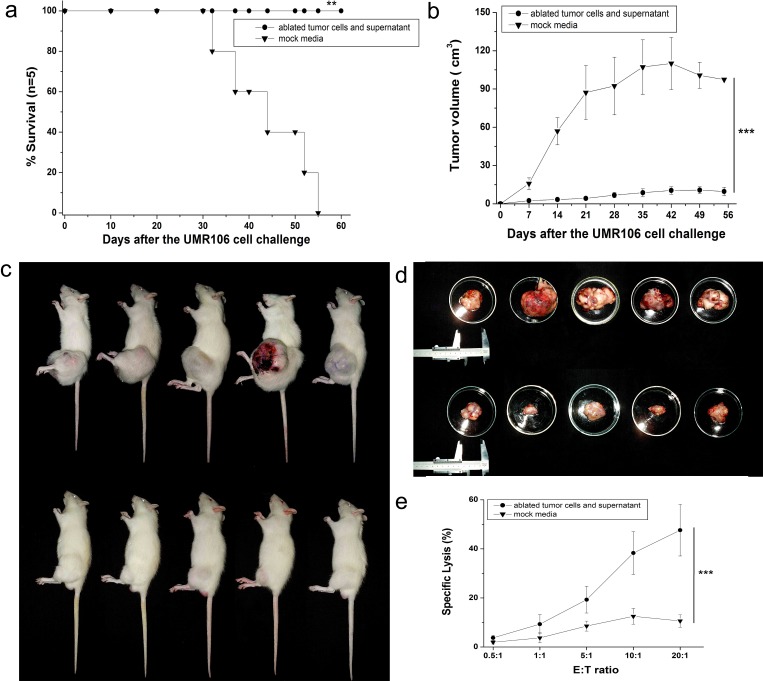
Complete protection of rats against lethal challenge with osteosarcoma cells (a) Survival curve of vaccinated mice and mock media control after tumor challenge. All ablated tumor cells/supernatant-vaccinated rats survived after tumor challenge and appeared to be tumor-free by log-rank test compared with the mock media control group. (b) After challenge with a lethal dose of 1×10^7^ osteosarcoma UMR106 cells, tumor growth was assessed every 7 days with a caliper, and tumor volume compared between ablated tumor cell-/supernatant-vaccinated mice and mock media control. Data are the mean ± SEM. (c) Representative images of growth of UMR106 tumor cells in the mock media control group (upper) and ablated tumor cells/supernatant-vaccinated group (lower). (d) Representative tumor images after killing and dissection in the mock media control group (upper) and ablated tumor cells/supernatant-vaccinated group (lower). (e) Splenocytes isolated from ablated products-vaccinated mice also showed a strong cytotoxic T-cell effect against fresh UMR106 cells *in vitro*, which was significantly different from that seen in the mock media control group. *P<0.05, **P<0.01, ***P<0.001.

Immunization with ablated tumor cells/supernatant proved significantly more efficient than mock media controls in eliminating implanted tumor cells (*P*<0.01). All vaccinated rats were able to reject tumor challenge and remained tumor-free for >8 weeks, whereas rats from the mock media control group developed tumors and died within 8 weeks (Fig. 3a). Tumor volume was also compared every week between vaccinated rats and the control group, and mock media-immunized rats showed much larger tumor volumes than ablated products-immunized rats (Figs 3b–d, *P*<0.001). The cytotoxicity of splenocytes isolated from ablated products-vaccinated mice or mock media control group also showed significant differences (Fig. 3e, *P*<0.001). These results were consistent with our finding from the *in vivo* mouse experiment that integration of ablated tumor cells and ablated supernatants was crucial for MWA products to elicit ICD.

### MWA induces immunogenic osteosarcoma cell death *in situ*

To investigate the induction of antitumor immune responses after tumor destruction *in situ*, we developed a rat UMR106 model in which microwaves ablated established tumors. We optimized tumor size at the moment of ablation, duration of ablative cycles, and impedance. Two consecutive treatment cycles of 80 s with an impedance of 400 Ω covering the entire tumor area (mean radius: 2 cm) yielded the best results.

CRT exposure on the cell surface of UMR106 cells was assessed after MWA for 10, 20 or 30 min or after mock ablation (0 min) by immunofluorescence staining followed by microscopic examination. After indicated treatments, cells were stained with rabbit anti-CRT antibody (or an isotype-matched control antibody; data not shown) and counterstained with goat anti-rabbit antibody (Alexa 488). Nuclei were stained with Hoechst 33258.

**Figure 4:**
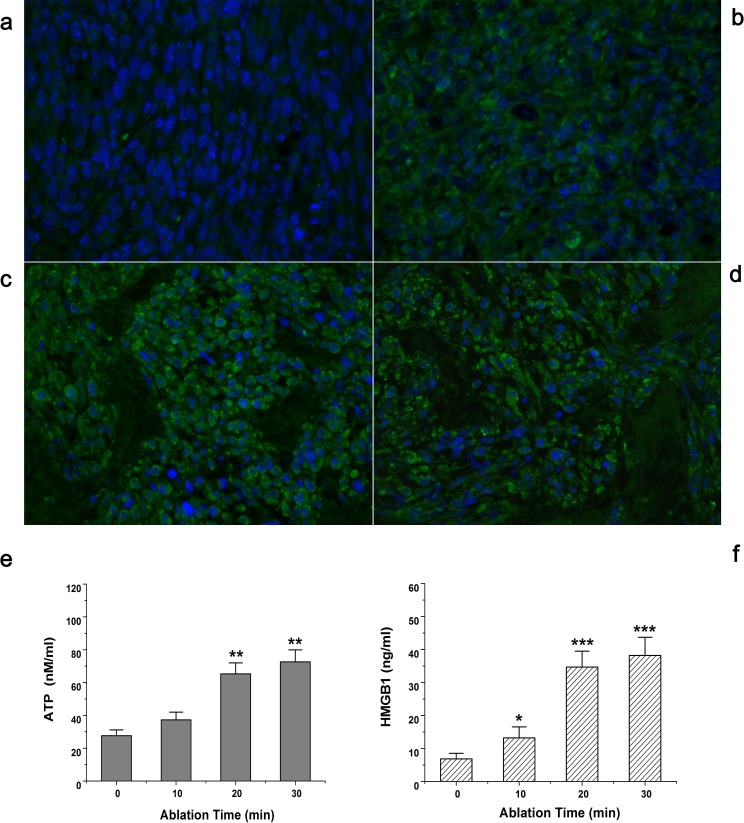
Exposure of tumors to MWA *in situ* increased expression of CRT and release of ATP and HMGB1 SD rats bearing UMR106 transplanted tumors were exposed to time-dependent MWA (10, 20, 30 min) or left untreated (0 min). After 24 h, tumors were removed surgically, and evaluated by immunofluorescence staining (40×) for CRT expression. (a) Mock ablation (0 min) failed to elicit CRT exposure. (b) MWA for 10 min showed only weak and uneven fluorescence signals. (c) After treatment with MWA for 20 min, CRT expression was much increased. (d) MWA for 30 min showed strong fluorescence signals in tumor tissue but visible fluorescent fragments suggest lysis of tumor cells. (e) MWA *in situ* induced time-dependent ATP release into peripheral blood. (f) HMGB1 secretion also demonstrated time-dependent effects after MWA *in situ*. *P<0.05, **P<0.01, ***P<0.001.

CRT exposure determines the immunogenicity of MWA-induced cell death [8]. Immunofluorescence analyses of osteosarcoma 24 h after ablation *in situ* showed increased expression of CRT. In identical conditions, mock-ablated UMR106 tissue failed to elicit pre-apoptotic CRT exposure (Fig. 4a). However, when ablation occurred, CRT expression was upregulated gradually. Translocation was also shown to be time-dependent. MWA for 10 min began elicited green fluorescence signals, but they were quite weak and distributed unevenly (Fig. 4b). After treatment with MWA for 20 min or 30 min, CRT expression on tumor tissue was increased considerably (Figs 4c–d). However, more fluorescent fragments appeared on slides after MWA for 30 min, suggesting lysis of tumor cells. Exposure to MWA *in situ* could also increase the release of DAMPs into peripheral blood. Exposure to MWA for 20 min induced significant release of ATP, which was approximately in accordance with the effects obtained from MWA for 30 min (Fig. 4e). In contrast, mock ablation (0 min) could not elicit the same level of ATP release (*P*<0.01). HMGB1 secretion also demonstrated similar time-dependent effects (Fig. 4f, *P*<0.001).

### MWA exposure *in situ* causes histologic changes and increases in expression of CD8^+^ T cell populations in rat osteosarcoma tissue

**Figure 5:**
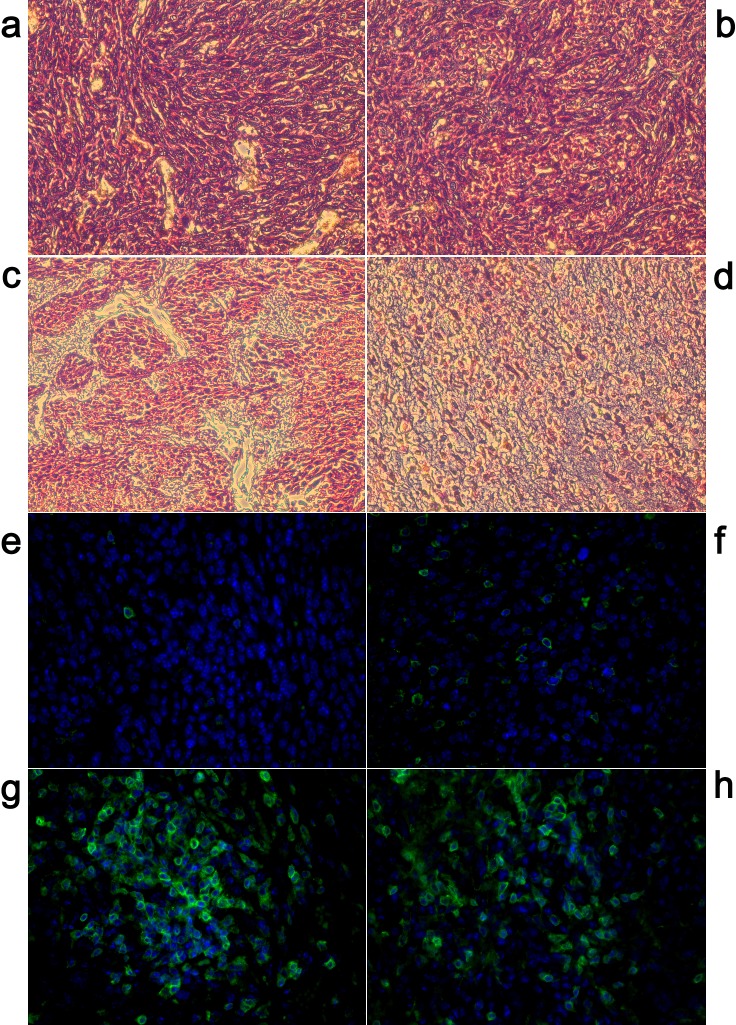
Exposure of tumors to MWA *in situ* showed histologic changes and increases in CD8 population in rat osteosarcoma tissue H&E staining of ablated tumor tissues (40×). (a) In the mock-ablated control group, tumor cells displayed a large nucleus surrounded by a well-defined cytoplasm and cell membrane. (b) After treatment with MWA for 10 min, tumor cells began to show gradual contraction, and a small nucleus and large extracellular matrix were observed. (c) Most tumor cells in the tissue ablated for 20 min underwent pyknosis, more inflammatory cells were recruited into the ablation field, and a wide range of necrosis of tumor cells did not occur. (d) Upon MWA for 30 min, tissue necrosis was visible and continued increase in cellular eosinophilia, vascular congestion and infiltration of inflammatory cells observed. (e) Mock ablation (0 min) failed to elicit recruitment of CD8^+^ T cells'. (f) MWA for 1 0min generated green fluorescence signals, revealing CD8^+^ T cells to be gradually recruited into the ablation field. (g–h) Upon MWA for 20 min or 30 min, considerable infiltration of CD8^+^ T cells in tumor tissue was observed, suggesting MWA-induced immunologic cellular immunologic responses.

Histologic examination of tissue taken from ablated tumor sites was undertaken 24 h after ablation *in situ*. H&E staining was employed to observe changes in tumor microenvironment. Microscopically, mock-ablated UMR106 tumor cells were characterized by bulky fusiform or triangular cells along nutrient vessels with nuclear pleomorphism, mitoses, bone invasion and necrosis (Fig. 5a). After MWA for 10 min, tumor cells began to contract gradually, and a smaller nucleus and larger extracellular matrix was evident (Fig. 5b). Upon MWA for 20 min: localized tissue necrosis became evident; more inflammatory cells were recruited into the ablation field; the remaining tumor cells showed an amorphous and irregular appearance; nucleus and cytoplasm shrank (Fig. 5c). After exposure to MWA for 30 min: karyopyknosis and nuclear fragmentation were present in most ablated tumor cells; cell contours were ruptured; considerable infiltration of inflammatory cells was observed (Fig. 5d).

To ascertain if cellular immunologic responses contributed to the major anti-tumor effects in the microwave-ablated field, immunofluorescence analyses was undertaken to detect infiltration of CD8^+^ T cells. After indicated treatments, cells were stained with rabbit anti-CD8 antibody (or an isotype-matched control antibody; data not shown) and counterstained with the goat anti-rabbit Alexa 488. Nuclei were stained with Hoechst 33258. Under a fluorescence microscope, mock ablation (0 min) failed to elicit recruitment of CD8^+^ T cells (Fig. 5e). MWA for 10 min generated visible green fluorescence signals, revealing CD8^+^ T cells to be recruited gradually into the ablation field (Fig. 5f). Upon MWA for 20 min or 30 min, considerable infiltration of CD8^+^ T cells in tumor tissue was observed, suggesting MWA-induced cellular immunologic responses (Figs 5g–h).

### CD8^+^ T cells contribute to the immunogenicity of MWA-induced cell death, and ICD triggered by Fas-FasL binding has a fundamental role in the killing of osteosarcoma cells by vaccine-elicited CD8^+^ T cells

To determine further the types of vaccine-elicited immune responses needed for protection, we undertook T-cell depletion experiments using the commercial monoclonal antibodies anti-CD4 and anti-CD8 to deplete CD4^+^ and CD8^+^ T cells, respectively. T-cell depletion was done one day before tumor challenge and repeated every week to maintain mice in a depleted state. In all cases, T-cell depletion was confirmed by FACS analyses of PBMCs (data not shown).

**Figure 6:**
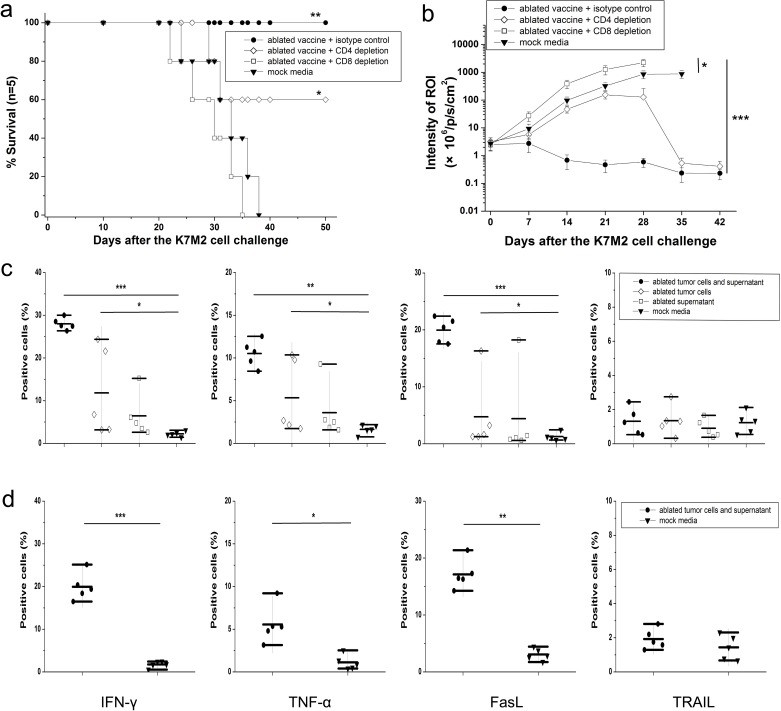
Mechanism of killing of osteosarcoma cells induced by vaccine-elicited CD8^+^ T cells (a) Survival curve showing that vaccinated mice failed to reject K7M2 tumor challenge after depletion of CD8^+^ T cells. Partial protection was achieved with 60% of cells surviving rate after depletion of CD4^+^ T cells. (b) After challenge with a lethal dose of K7M2 cells, tumor growth was assessed by bioluminescence imaging at day 42. When CD8^+^ T-cell depletion was carried out, tumors of vaccinated mice showed more aggressive growth. When PBS was used instead of anti-CD8 monoclonal antibody, vaccinated mice still proved to be very efficient in eliminating implanted tumor cells. (c) Mice immunized with ablated tumor cells/supernatant released significantly higher levels of IFN-γ and TNF-α, and showed much higher level of FasL expression on CD8^+^ T cells than other control groups. (d) Similar results were observed when culturing splenocytes from vaccinated rats with UMR106 cells. *P< 0.05, **P<0.01, ***P<0.001.

Repeatedly, ablated products-immunized mice were able to eliminate tumor growth effectively to achieve 100% survival. Partial protection was observed after depletion of CD4^+^ T cells, resulting in 60% survival. After depletion of CD8^+^ T cells, vaccinated mice failed to reject K7M2 tumor challenge, and all animals died within 5 weeks (Fig. 6a). The ROI of tumor-bearing mice was compared every week between vaccinated mice with or without CD8^+^/CD4^+^ T-cell depletion. Ablated products-vaccinated mice injected with isotype control (purified rat IgG) instead of CD8+/CD4+ T cell-blocking antibodies were extremely efficient in eliminating implanted tumor cells whereas, when CD8^+^ T-cell depletion was undertaken, the tumors of vaccinated mice showed more aggressive growth (Fig. 6b, *P*<0.001). Depletion of CD4^+^ T cells failed to generate the same level of antitumor protection as that seen in the isotype control group, and only some animals remained tumor-free. The tumor protection obtained was not due to antibody-dependent cellular cytotoxicity because passive transfer of antisera derived from ablated products-immunized mice did not show protective effects in SCID mice (data not shown). Taken together, CD8^+^ T cells seemed to be essential for eliminating osteosarcoma cells.

To determine the functionality of T cells, splenocytes of vaccinated mice were co-cultured with K7M2 cells *in vitro* and then intracellular contents stained with anti-IFN-γ and anti-TNF-α. Mice immunized with ablated tumor cells/supernatant released significantly higher levels of IFN-γ and TNF-α compared with ablated tumor cells alone, ablated supernatant alone, or mock media controls. Next, we investigated the apoptosis pathways involved in CD8^+^ T cell-mediated killing. Ablated tumor cells/supernatant-vaccinated mice showed much higher level of FasL expression on CD8^+^ T cells than the other control groups. In contrast, no significant differences were found with TRAIL expression on CD8^+^ T cells between vaccinated mice and any control group (Fig. 6c, *P*>0.05). Similar results were observed when culturing splenocytes from vaccinated rats with UMR106 cells, and CD8^+^ T cells released more functional cytokines, including IFN-γ (*P*<0.001) and TNF-α (*P*<0.01). Expression of FasL expression in rat-derived CD8^+^ T cells was in accordance with the results obtained from murine splenocytes, and TRAIL expression remained at quite a low level and was not significantly different (Fig. 6d).

## DISCUSSION

Classical cancer chemotherapies seem to engage the immune system, and part of their efficacy might result from this engagement. For example, chemotherapeutic agents such as anthracyclines and OXP induce tumor cells to undergo apoptosis, which is associated with cell-surface exposure of CRT. We made similar observations in experiments with the MWA-induced osteosarcoma cells. These findings support our premise that, after therapeutic microwave ablation, residual viable tumor cells can also work as “tumor vaccines” and induce effective antitumor immune responses by upregulating expression of CRT and exploiting the immunostimulatory environment in multiple carcinomas.

We report here that MWA induces the cardinal signs of ICD (upregulation of expression of CRT; release of ATP and HMGB1) in multiple osteosarcoma types. The longest ablation time used here (30 min) is almost that used in the clinic and was selected to ensure death of all cells involved in the ablation range. However, the ATP/HMGB1 secretion and CRT expression we observed at a sublethal time interval (20 min) suggests that ICD may also be achieved under this ablation condition, and with greater cell viability. Conversely, use of the appropriate temperature will enable the heated necrotic bone to maintain its osteoconductive and osteoinductive abilities, which will benefit the rebuilding of new bone [16].

This concept is supported further by our findings when tumor ablation was carried out *in situ*. During the past 20 years, >1000 patients with malignant or highly aggressive bone tumors of the extremities or pelvis have been treated in our department by separation of tumor-bearing bone and heat necrosis *in situ* [6]. This method maximizes retention of the basic functions of treated tumor-bearing bone: supporting body weight and providing muscle attachment to move the body. Results from oncologic and functional viewpoints were encouraging, and drove us to continue with this approach to treat bone tumors.

MWA destroys tumors through application of a high temperature produced by electromagnetic energy to rapidly rotate adjacent polar water molecules within the targeted diseased tissue. This action leads to protein denaturation, cell-membrane disruption and, finally, coagulative necrosis with cellular death [17]. However, tumor cells from the most outer margin of the effective ablation range could not be confirmed to suffer such protein denaturation. To protect adjacent organs, the boundary temperature should maintained at ≤50°C and the ablation time must be monitored strictly.

In the present study, we focused on the residual tumor cells from the gray zone of ablation. The *in situ* ablation model is very important for mimicking the therapeutic process. UMR106 is a very aggressive, poorly immunogenic osteosarcoma cell line that frequently metastasizes to the lungs. Originally, it was derived from a rat-transplantable osteogenic sarcoma with an osteoblastic phenotype [18]. According to our previous studies [19–20], this model of rat osteosarcoma is quite stable and reproducible. The mean radius of implanted tumors was 30.46±7.82 mm, which was suitable for insertion of one needle antenna and receipt of thermal energy during ablation. After *in situ* ablation, CRT expression on the tumor cell surface was upregulated significantly. Expression of CRT on the tumor cell surface might contribute to the capture of apoptotic bodies by dendritic cells (DCs) and so might elicit tumor-specific CD8^+^ T cell-mediated immune responses. These T cells might contribute to the elimination of tumor cells that have not been ablated directly by hyperthermia, and also protect the host from tumor recurrence or invasion of new tumor cells.

Some investigators have reported regression of tumors at other anatomic site after curative heating of the primary tumor: the “abscopal effect” [21]. In our opinion, the most rational explanation of the abscopal effect is the concept of ICD. One of the major effects ICD can unleash an effective immune response is induction of a strong “danger signal” generated by damaged tissue under endoplasmic reticulum stress [22]. Dying tumor cells succumbing to ICD induce danger signals called “alarmins” that have immunogenic properties. Various mechanisms with different peptides, cytokines, and cells are involved in this process to prevent tumor recurrence, and can occur even at other anatomic sites. For example, translocation of CRT from the endoplasmic reticulum to the cell surface can enhance presentation of apoptotic cell antigens to antigen-presenting cells (APCs) [23]. ATP is released to recruit and activate DCs [24]. HMGB1 might also be released and act as a “neo-antigen”, initiating an inflammatory response through binding toll-like receptor-4 on DCs [25]. Our results from *in vitro* and *in situ* experiments are consistent with cardinal signs through ICD. Hence, one could conclude that sublethal MWA can achieve tumor elimination through the initiation of ICD.

Another interesting issue in ICD research is to confirm links between exposed DAMPs and effector cells *via* activation of DCs. After sufficient ablation (>20 min), immunofluorescence staining results revealed CRT exposure in tumor tissue to be increased significantly. To compare the severity of the local inflammatory response, CD8^+^ T cells were also stained for immunofluorescence analyses when infiltrating into tumor tissues. Many vaccine-elicited CD8^+^ T cells were recruited into tumors and could retain their effector functions to remove residual tumor cells. These two results were approximately correlated, so CRT exposure might be one of the causes of recruitment of CD8^+^ T cells. Subsequent studies were done to detect the molecular mechanisms responsible for CD8^+^ T cell-mediated killing. Vaccinated mice showed much higher levels of FasL expression on CD8^+^ T cells than that seen in control groups. In contrast, no significant differences were found for TRAIL expression on CD8^+^ T cells between vaccinated mice and control groups. However, this was only a preliminary exploration of the mechanism of the killing of activated CD8^+^ T cells: the downstream signaling events activated by Fas/FasL will be assessed in future experiments.

We believe that MWA-induced tumor vaccines have important advantages. First, the tumor necrosis caused by microwave ablation can lead to the release of various tumor-associated antigens, which might be processed and presented by DCs to T cells, resulting in *de novo* activation of tumor-specific immunity. This type of antitumor immune response could be more intensive than that seen in antigen-speciﬁc cytotoxic T cells, which recognize only a fraction of the epitopes expressed on tumor cells. Second, denatured protein molecules from coagulative necrosis by ablation could also enhance the local inflammatory response [26]. Use of ablated tumor vaccines can also bypass the requirement for defined MHC alleles and for expression of identified antigens by tumors. Immunogenic whole tumor cells can encode multiple epitopes and recruit a wide spectrum of T-cell clones [27], among which CD8^+^ cytotoxic T cells are the dominant population.

## MATERIALS AND METHODS

### Ethical approval of the study protocol

Experimental procedures involving animals were conducted under a protocol reviewed and approved by the Ethics Committee of Tangdu Hospital, Fourth Military Medical University (permit number: 2012036; Xi'an, China). All efforts were made to minimize the number of animals used and to ameliorate their suffering.

### Animals and cell lines

Female Balb/c mice and Sprague–Dawley (SD) rats (mean age, 4 weeks) were purchased from the Laboratory Animal Research Center of the Fourth Military Medical University. They were fed under specific pathogen-free conditions. K7M2 syngeneic to Balb/c mice, UMR106 syngeneic to SD rats, and MG63 human osteosarcoma cells were purchased from American Type Culture Collection (Manassas, VA, USA). Cell lines were maintained in RPMI 1640 medium supplemented with 10% fetal bovine serum (FBS) (Gibco, Billings, MT, USA), 2 mM L-glutamine (Gibco) and 1% penicillin/streptomycin antibiotics (Gibco) at 37°C in an atmosphere of 5% CO_2_.

### Design and generation of a luciferase-expressing osteosarcoma cell line

A *BamH*I-*EcoR*I fragment of luciferase was amplified by polymerase chain reaction from the pGL3-basic vector (Promega, Fitchburg, WI, USA) and inserted into retroviral vector pBABE-puro to generate pBABE-luc-puro. Using a polyethylenimine transfection reagent, the modified pBABE-luc-puro vector was transfected together with the packaging plasmid pCL-Amphotropic into 293T cells to generate a retroviral vector supernatant. K7M2 mouse osteosarcoma cells were then infected with the retroviral vector and selected with 1 μg/mL puromycin. Single clones were obtained by limiting dilution of puromycin-resistant cells and screened under the Xenogen IVIS® 100 Imaging system (PerkinElmer, Waltham, MA, USA). Positive clones were selected and grown in duplicate to obtain K7M2–luciferase cells.

### Bioluminescence imaging (BLI)

BLI was undertaken with the Xenogen IVIS 100 Imaging system. Bioluminescent substrate firefly D-Luciferin (PerkinElmer) was dissolved in phosphate-buffered saline (PBS) to prepare a 200× luciferin stock solution (30 mg/mL). For BLI of cells grown *in vitro*, the substrate solution was obtained from a 1:200 dilution of D-Luciferin stock solution in complete medium at a final concentration of 150 μg/mL. Culture medium was aspirated from cells before addition of substrate solution. Samples were then placed into the imaging chamber to collect the bioluminescent signal (exposure time: 10–30 s). Regions of interest (ROI) were created to encompass the area with most intense light. Signal intensity (photons/s/cm^2^/sr) was calculated using Living Imaging v3.2 (PerkinElmer). For BLI of tumor growth *in vivo*, mice were anesthetized (ketamine, 100 mg/kg, i.p.; xylazine, 10 mg/kg, i.p.) before D-luciferin was administered *via* the intraperitoneal route to deliver a dose of 150 mg/kg. Mice were then placed in the imaging chamber and left for 5 min for substrate distribution. Images were taken (exposure time: 30–120 s) and analyzed as described above.

### Microwave ablation

The microwave machine (KY-2000; Kangyou Medical, Nanjing, China) used in this study comprised four parts. The first part was eight microwave generators, each of which produced 100 W at 2450 ± 20 MHz. Eight coaxial cables were used for transmitting energy. The second part was eight needle antennae (diameter: 1.9 mm (15 G); length, 18 cm). The third part was a temperature-monitoring system: eight probes continuously measured temperature in real time during ablation. The final part was software controlling the energy output of the preset temperature.

For *in vitro* ablation, solutions of K7M2 cells, UMR106 cells, and MG63 cells were adjusted to 1×10^7^/mL. Then, 1 mL of each sample was collected into a 1.5-mL microcentrifuge tube and exposed to one needle antenna (which provided energy delivery). The temperature on the antenna tips was set at 50°C, and ablation lasted for 0, 10, 20, and 30 min. Heated cells and supernatant were collected for detection or immunization.

For *in vivo* ablation, tumor-bearing rats were anesthetized (ketamine, 100 mg/kg, i.p.; xylazine, 10 mg/kg, i.p.) and shaved at the tumor area and ipsilateral flank. The tumor area was disinfected with alcohol. A MWA needle antenna was inserted subcutaneously in the center of the tumor. After needle placement, impedance was evaluated on the microwave generator system. Treatment was started by delivering microwave energy. During MWA, the temperature was monitored using thermal monitoring probes. Treatment was considered successful if the temperature at the tumor margin was 50°C for the indicated time. All procedures were carried out under strictly sterile conditions. After the procedures, the incision was closed routinely, and rats kept separately in normal housing conditions. All procedures were carried out by the same surgeon.

### Immunization with ablated vaccine and tumor challenge

Groups of five mice or rats were immunized with 1×10^6^ ablated K7M2 tumor cells or 1×10^7^ ablated UMR106 tumor cells combined with 1 mL of ablated supernatant. This mixture was delivered subcutaneously with a needle into the mouse's right flank or the rat's groin thrice at two-week intervals. Then, vaccinated animals were challenged subcutaneously on their left flank with a lethal dose of tumor cells (1×10^6^ of K7M2 or 1×10^7^ of UMR106) and monitored for tumor growth and survival every week. After the animals had been killed at the indicated time point, splenocytes were collected for analyses of the immune response.

### Histologic and morphometric procedures

Histologic examination of tissues taken from the site of tumor implantation was undertaken by a very experienced pathologist. Ablated tumor tissues were fixed in 10% buffered formalin solution for 24 h, dehydrated in ascending grades of ethanol, and embedded in paraffin. Serial sections (thickness, 5 μm) were cut for at least three temperature-measured sites from each sample. Some sections were stained for general morphology using hematoxylin and eosin (H&E) and slides examined under a light microscope (BX-50; Olympus, Tokyo, Japan) and photographed with a digital camera (Olympus).

For immunofluorescence stains, rehydrated sections were pretreated with Triton-X100 for 15 min, incubated with rabbit polyclonal antibodies to CRT (Abcam, Cambridge, UK) or mouse monoclonal antibodies to CD8 (Abcam) at 1:100 dilutions for 1 h, rinsed thrice with PBS for 5-min each, and incubated with goat polyclonal secondary antibody to rabbit IgG (Alexa Fluor® 488, Abcam) or goat polyclonal secondary antibody to mouse IgG (Alexa Fluor® 488) at 1:100 dilutions for 1 h. After rinsing thrice with PBS, nuclei were stained with Hoechst 33258 (Sigma-Aldrich, St Louis, MO, USA). Staining was visualized at 488 nm using an inverted microscope equipped with a digital camera (Olympus).

### T-cell depletion *in vivo*

For *in vivo* T-cell depletion experiments, mice were injected with murine anti-mouse CD4 (500 mg, i.p.; Bio X Cell, West Lebanon, NH, USA) or murine anti-mouse CD8 (Bio X Cell), or isotype control (Rat IgG2b, Bio X Cell). The first injection was given one day before tumor challenge, followed by multiple injections at seven-day intervals. In all cases, T-cell depletion was confirmed by flow cytometric analyses of peripheral blood mononuclear cells (PBMCs).

### Sample preparation for flow cytometric analyses

Ablated tumor cells or splenocytes (5×10^5^) were prepared for immunostaining for fluorescence-activated cell sorting (FACS) analyses. Anti-mouse calreticulin-phycoerythrin (PE) and matched isotype control antibody were obtained from R&D Systems (Minneapolis, MN, USA). Anti-mouse Fas ligand–fluorescein isothiocyanate (FasL-FITC) and tumor necrosis factor-related apoptosis-inducing ligand (TRAIL)-PE were from BD Biosciences (Franklin Lakes, NJ, USA). For staining of surface markers, cells were washed with FACS staining buffer (PBS containing 0.2% FBS) and resuspended in 100 μL of staining buffer containing the corresponding antibodies, followed by incubation at 4°C for 1 h. After washing twice with FACS staining buffer, cells were fixed in 200 μL of 1% paraformaldehyde.

For intracellular staining of interferon (IFN)-γ and TNF-α, after surface staining with anti-mouse CD3-AF700, CD4-V450, and CD8-PerCP antibodies (BD Biosciences), cells were permeabilized in 100 μL of Fixation/Permeabilization Solution (BD Biosciences) for 20 min at 4°C, washed with Permeabilization/Washing buffer (BD Biosciences), and then underwent intracellular staining with anti-IFN-γ-PE and anti-TNF-α-FITC (BD Biosciences). Cellular fluorescence was examined on a FACSCalibur instrument (BD Biosciences) and analyzed using CellQuest (BD Biosciences).

### Statistical analyses

Data are the mean ± standard deviation. Significant differences between time points or groups were analyzed using ANOVA for repeated measures with Tamhane's T2 method for multiple comparisons employing SPSS v17.0 (SPSS, Chicago, IL, USA). P<0.05 was considered significant.

### Abbreviations

MWA, microwave ablation; RFA, radiofrequency ablation; ICD, immunogenic cell death; FBS, fetal bovine serum; BLI, bioluminescence imaging; ROI, regions of interest; H&E, hematoxylin and eosin; PBMCs, peripheral blood mononuclear cells; FACS, fluorescence-activated cell sorting; TRAIL, tumor necrosis factor-related apoptosis-inducing ligand; OXP, oxaliplatin; DAMPs, damage-associated molecular patterns; CRT, calreticulin; HMGB1, high-mobility group box 1; ATP, adenosine triphosphate; MHC, major histocompatibility complex; DCs, dendritic cells; APCs, antigen-presenting cells; PE, phycoerythrin; PBS, phosphate-buffered saline; IFN, interferon.

## CONCLUSION

The present study provided evidence that MWA-processed tumor cells could be applied to induce specific antitumor effects, especially for *in situ* ablation. Whole ablated tumor cells might serve as general cancer antigens, and hyperthermia-induced exposure to the composition of whole tumor cells might be a superior alternative to other well-known antigen-loading vaccines [28]. This study provided the foundations for an effective and broadly applicable treatment to a wide range of cancer indications for which tumor-associated antigens have not been identified. MWA could be used in combination with immunotherapy, especially for patients who have failed chemotherapy or have limited treatment options.
